# Adrenal Mass Characterization in the Era of Quantitative Imaging: State of the Art

**DOI:** 10.3390/cancers14030569

**Published:** 2022-01-23

**Authors:** Maxime Barat, Anne-Ségolène Cottereau, Sébastien Gaujoux, Florence Tenenbaum, Mathilde Sibony, Jérôme Bertherat, Rossella Libé, Martin Gaillard, Anne Jouinot, Guillaume Assié, Christine Hoeffel, Philippe Soyer, Anthony Dohan

**Affiliations:** 1 Department of Radiology, Cochin Teaching Hospital, AP-HP, Université de Paris, 75014 Paris, France; maxime.barat@aphp.fr (M.B.); philippe.soyer@aphp.fr (P.S.); 2 Faculté de Médecine, Université de Paris, 75006 Paris, France; annesegolene.cottereau@aphp.fr (A.-S.C.); sebastien.gaujoux@aphp.fr (S.G.); mathilde.sibony@aphp.fr (M.S.); jerome.bertherat@aphp.fr (J.B.); rossella.libe@aphp.fr (R.L.); martin.gaillard@aphp.fr (M.G.); anne.jouinot@aphp.fr (A.J.); guillaume.assie@aphp.fr (G.A.); 3 Department of Nuclear Medicine, Cochin Hospital, AP-HP, 75014 Paris, France; florence.tenenbaum@aphp.fr; 4 Department of Pancreatic and Endocrine Surgery, Pitié-Salpetrière Hospital, AP-HP, 75013 Paris, France; 5 Department of Pathology, Cochin Hospital, AP-HP, 75014 Paris, France; 6 Department of Endocrinology, Cochin Hospital, AP-HP, 75014 Paris, France; 7 Department of Digestive, Hepatobiliary and Endocrine Surgery, Cochin Hospital, AP-HP, 75014 Paris, France; 8 Department of Radiology, Hôpital Robert Debré, 51092 Reims, France; choeffel-fornes@chu-reims.fr

**Keywords:** adrenal glands, adrenal glands neoplasm, adrenal gland disease, tomography, X-rays computed, magnetic resonance imaging, positron emission tomography, artificial intelligence

## Abstract

**Simple Summary:**

Non-invasive characterization of adrenal lesions requires a rigorous approach. Although CT is the cornerstone of adrenal lesion characterization, a multimodality multiparametric imaging approach helps improve confidence in adrenal lesion characterization.

**Abstract:**

Detection and characterization of adrenal lesions have evolved during the past two decades. Although the role of imaging in adrenal lesions associated with hormonal secretion is usually straightforward, characterization of non-functioning adrenal lesions may be challenging to confidently identify those that need to be resected. Although many adrenal lesions can be readily diagnosed when they display typical imaging features, the diagnosis may be challenging for atypical lesions. Computed tomography (CT) remains the cornerstone of adrenal imaging, but other morphological or functional modalities can be used in combination to reach a diagnosis and avoid useless biopsy or surgery. Early- and delayed-phase contrast-enhanced CT images are essential for diagnosing lipid-poor adenoma. Ongoing studies are evaluating the capabilities of dual-energy CT to provide valid virtual non-contrast attenuation and iodine density measurements from contrast-enhanced examinations. Adrenal lesions with attenuation values between 10 and 30 Hounsfield units (HU) on unenhanced CT can be characterized by MRI when iodinated contrast material injection cannot be performed. ^18^F-FDG PET/CT helps differentiate between atypical benign and malignant adrenal lesions, with the adrenal-to-liver maximum standardized uptake value ratio being the most discriminative variable. Recent studies evaluating the capabilities of radiomics and artificial intelligence have shown encouraging results.

## 1. Introduction

Adrenal lesions are present on 4–6% of routine abdominal imaging examinations, mostly as incidental adrenal lesions consisting of asymptomatic adrenal lesions discovered when performing an abdominal imaging examination for any other indication, with the exclusion of adrenal lesions discovered during the screening of patients with hereditary syndromes or extra-adrenal tumors [1]. Adrenal lesions are a heterogeneous group of conditions with different clinical presentations and prognoses [1]. Most adrenal lesions are benign; approximately 70% are adrenal adenomas (AA) and 20% consist of other benign lesions such as myelolipomas, nodular hyperplasia and pheochromocytomas, and other rare histological lesions including, but not exclusively, hematoma, ganglioneuroma, lymphangioma, schwannoma and vascular malformation. Less than 10% of adrenal lesions are malignant, consisting of metastases mostly from lung, kidney, breast, melanoma and gastrointestinal tract cancers and, more rarely, adrenocortical carcinoma (ACC), which represent 1% of all adrenal incidental lesions, as well as other rarer conditions such as primary adrenal lymphoma, sarcoma or rare malignant pheochromocytoma [2,3]. Although the role of imaging in the diagnosis of adrenal lesions in patients with the associated increased function is usually straightforward, the characterization of incidental adrenal lesions to confidently identify those that need to be removed is more challenging. 

The objective of this review article was to provide an overview of the essentials of adrenal gland imaging, including the latest advancements and newest imaging techniques.

## 2. Genetic Syndromes

Inherited genetic disorders represent major risk factors for adrenal lesions. Pheochromocytomas are present in several genetic syndromes [4,5], which include Von-Hippel–Lindau syndrome; multiple endocrine neoplasia Type 2 with the associated thyroid medullary carcinomas and hyperparathyroidism for Type 2a, or neuromas and a marfanoid appearance for Type 2b; Type 1 neurofibromatosis syndrome with multiple neurofibromas; and succinate dehydrogenase genetic syndromes (i.e., SDHB and SDHD) [4,5]. Most of the implicated genes are tumor suppressor genes [4,5]. Adrenocortical genetic syndromes are less well known but several mutations with familial forms have been described such as KCNJ5, ATP1A1, ATP2B3 and CACNA1D mutations in aldosterone-producing AA; PRKACA mutations in cortisol-producing AA; ARMC5 mutations in primary bilateral macronodular adrenocortical hyperplasia (PBMAH) and ZNRF3 mutations in ACC [6]. Primary pigmented nodular adrenocortical disease (PPNAD) is a form of bilateral adrenocortical hyperplasia, with independent ACTH cortisol secretion associated with several possible mutations [6]. Non-nodular adrenal lesions may also be associated with genetic syndromes, the most frequent being 21-hydroxylase deficiency [7]. 

## 3. Normal Adrenal Glands on Imaging

Normal adrenal glands are paired retroperitoneal glands located above the kidney and are included in the renal fascia, displaying a flipped “V” shape. Briefly, their parenchyma is divided into two parts: the cortex, which normally secrets cortisol, aldosterone and sexual hormones, and the medulla, which produces catecholamines [2]. The mean normal adrenal gland volume was reported to be 8.5 ± 1.40 mL but can be affected by various factors such as weight and sex [8]. Normal adrenal glands have a signal and density close to those of the liver and spleen, homogeneous enhancement and a mean standardized uptake value (SUV) of 2.3 ± 0.7 (mean ± SD), and an adrenal-to-liver maximum SUV ratio of 1.0 ± 0.3 on ^18^F-fluorodesoxyglucose (FDG) positron emission tomography (PET)/CT [9]. 

## 4. Adrenal Hyperplasia

Adrenal hyperplasia (AH) is a common cause of morphological adrenal irregularities. AH can be non-nodular (defined as an adrenal body thickness of >10 mm and an adrenal limb thickness of >5 mm with convex borders, without any measurable adrenal nodules) or nodular (confluent adrenal irregularities giving a lumpy appearance separated by atrophic adrenal tissue) [10,11]. Etiologies include adrenal and extra-adrenal diseases (Table 1). AH can be associated with secretion, mostly cortisol secretion, which is adrenocorticotropic hormone (ACTH)-dependent in 80–85% of AH cases [11]. Moreover, smooth enlargement or nodularity of the adrenal glands is associated with older age and may also represent a hyperplastic stress response to cancer [11]. In this regard, in a series of 197 patients with lung cancer, 11% of them had smoothly enlarged adrenal glands on CT that were not associated with later development of adrenal metastases [12]. Biological variables, clinical history and evolution help identify these causes more accurately than imaging examinations alone [13]. For the monitoring of patients with AH, three-dimensional (3D) volume assessments are more robust and more reproducible than two-dimensional imaging [14]. 

## 5. Other Non-Tumoral Adrenal Gland Abnormalities

In the specific situation of circulatory shock, the adrenal glands appear to be hyper-enhanced, defined as bilateral adrenal attenuation values greater than those of the inferior vena cava [15]. When quantifying this enhancement, an adrenal-to-spleen ratio of >1.41 in portal venous phase CT images is associated with a likelihood ratio of 41 for lethal outcomes [15]. The “hollow adrenal gland sign”, defined as peripheral enhancement during the arterial phase and whole-gland enhancement during the portal phase, is considered to be a specific pattern of septic circulatory shock compared with hemorrhagic shock [8]. Similarly, one study found that an adrenal volume of <10 cm^3^ was associated with the 28-day-mortality in patients with septic shock (hazard ratio, 0.014; 95% CI: 0.004–0.335) [16]. However, these findings were not confirmed in cardiogenic shock after resuscitation [17]. 

## 6. Typical Incidental Adrenal Lesions on Imaging

AA typically presents as a homogeneous adrenal nodule, with a largest diameter of <4 cm, regular margins and a mean attenuation value of ≤10 Hounsfield units (HU) on unenhanced CT or a signal drop on chemical-shift magnetic resonance imaging (MRI). They can be diagnosed with 78% accuracy, 55% sensitivity, 100% specificity, a positive predictive value (PPV) of 100% and a negative predictive value (NPV) of 65%, providing that the lesion is homogeneous and that the region of interest (ROI) is placed on the two-thirds of the lesion, excluding the cortex [18]. Similarly, adrenal cysts consist of a purely fluid-like lesion without enhancement, and myelolipoma is accurately characterized when a macroscopic fatty content is visible on either CT or MRI without any cut-off for the quantity of macroscopic fat required to establish the correct diagnosis [19]. Macroscopic fat has been reported in non-myelolipoma adrenal lesions such as ACC and renal metastasis, although very rarely, but no comparative studies exist yet; some authors have thus proposed the presence of 50% of macroscopic fat in the lesion to confidently diagnose myelolipoma [20]. Of note, characterization of small adrenal lesions has been poorly studied, so for asymptomatic and non-secreting adrenal lesions of <10 mm, the current guidelines do not recommend further exploration [1]. 

Thirty percent of AAs do not display the typical intracellular microscopical fatty content (i.e., the so-called “lipid-poor AAs”), and thus require image acquisition at 60 s and 10 min or 15 min after the start of intravenous administration of iodinated contrast material to further allow calculation of the absolute percentage washout (APW) and relative percentage washout (RPW) [21]. The 10-min delayed acquisition yields a lower accuracy than 15 min and may require confirmation with a 15 min acquisition if negative [21]. A cut-off of 60% for APW yielded 88% sensitivity and 89% specificity of differentiating between lipid-poor AA and non-adenoma lesions, and a cut-off of 40% for RPW yielded 100% sensitivity and 100% specificity [22,23]. Finally, a subset of non-AA lesions with varying malignant potential are incompletely characterized using conventional imaging and require further investigation for a definite diagnosis [10]. For the remaining adrenal lesions with indeterminate imaging features, the growth rate is a helpful criterion for distinguishing between benign and malignant lesions because only one-third of benign adrenal lesions grow, with a growth rate of ≤3 mm per year for all, whereas all malignant lesions grow at a rate of ≥5 mm per year [24]. 

For non-typical benign adrenal lesions and secreting lesions, or when surgery is considered, a multidisciplinary expert team meeting is recommended [1]. The major task for radiologists consists of discriminating between various possible adrenal lesions to allow best patient management, optimize treatment and avoid overtreatment [25]. 

## 7. Hyperfunctioning Adrenal Lesions

Approximately 50% of adrenal lesions secrete hormones in excess [1]. Hypercortisolism, hyperaldosteronism and catecholamine secretion are the three main secreting syndromes that can be associated with adrenal lesions, whereas androgen secretion is rare [1]. Secretions can be clinically significant or may be associated with biochemical abnormalities only, so that any adrenal lesion requires biological testing for cortisol and metanephrine secretion analysis [1]. Androgen secretion testing is restricted to patients with suspected ACC and aldosterone testing to patients with blood hypertension or hypokalemia [1]. The search for increased secretion is an important step because, except for patients with a contraindication, surgery is considered for hyperfunctioning adrenal lesions [1]. In addition, adrenal conditions associated with hyperfunctioning status are different from those without abnormal secretion [1]. 

### 7.1. Cortisol-Secreting Adrenal Lesions

Cortisol-secreting adrenal lesions (i.e., ACTH-independent) include AA and ACC. About 30% of AA and 90% of ACC cases secrete hormones [26]. In a prospective study, 98% (96/98) of ACCs had a size of >4 cm and 99% (97/98) had a non-contrast attenuation value of >20 HU [26]. A combined test strategy including imaging features with the size (>4 cm), non-contrast attenuation (>20 HU) and measurement of urine steroid metabolites was proposed and yielded 99% sensitivity and 80% specificity for differentiating between AA and ACC, and a NPV of 76% for the diagnosis of ACC [26]. 

### 7.2. Metanephrine-Secreting Adrenal Lesions

Up to 95% of pheochromocytomas secrete hormones in excess [4]. However, only 80% of catecholamine-secreting tumors are adrenal pheochromocytomas, whereas the remaining 20% are extra-adrenal paragangliomas, thus requiring an extensive search for the extra-adrenal locations when the adrenal glands have a normal appearance [27]. Although rare, non-secreting pheochromocytomas are associated with a greater prevalence of metastases than secreting ones [28]. Pheochromocytoma generally displays a heterogeneous pattern due to cystic or hemorrhagic changes [29]. A meta-analysis including 1217 pheochromocytomas found a size ranging from 12 to 150 mm and a mean unenhanced attenuation of 35.6 HU (range: 15.0–62 HU), all of which were >10 HU, even the heterogeneous ones [30]. However, on CT, despite a high degree of enhancement, 35% of pheochromocytomas had an APW and RPW similar to lipid-poor AA [29,31]. Pheochromocytomas demonstrating washout often have a peak enhancement greater than 110 HU [21]. Calcifications are present in 20% of pheochromocytomas [32]. On MRI, pheochromocytomas classically display hypersignals on T2-weighted images, but some authors found this pattern in only 50% of pheochromocytomas [33]. Diffusion-weighted imaging (DWI) has a role only for the detection of pheochromocytomas < 1 cm [34]. 

### 7.3. Aldosterone-Secreting Adrenal Lesions

Diagnosis and management of aldosterone-secreting adrenal lesions are important because appropriate treatment increases patients’ overall survival [35]. One study reported the similar diagnosis performance of imaging for the characterization of aldosterone-producing AAs and all AAs [36]. When compared with cortisol-producing AAs, aldosterone-producing AAs have a smaller size (24.50 ± 11.34 vs. 18.92 ± 7.98 respectively; *p* = 0.038) [37]. Finally, when there is doubt as to the responsible secreting gland because of bilateral adrenal lesions or bilateral hyperplasia, adrenal vein sampling may be required [38]. 

## 8. Hypofunctional States

Adrenal insufficiency (i.e., Addison’s disease) is a rare condition associated with a high mortality risk, which poses a diagnostic challenge [39]. Once pituitary diseases and genetics syndromes have been excluded, medical imaging, mostly CT, is used to evaluate the atrophic or enlarged status of both adrenal glands [39]. Atrophic glands are mostly related to autoimmune adrenalitis and rare genetic conditions, whereas enlarged ones may be due to hematoma, granulomatosis, primary tumors or metastasis [39]. 

## 9. Conventional Imaging to Differentiate between Benign and Malignant Adrenal Lesions

The most frequent malignant adrenal lesions are metastases. Their diagnosis is relatively straightforward during the follow-up of a patient with a known cancer, but an adrenal lesion may be the revealing manifestation of an unknown cancer in 2% of patients with an incidental adrenal lesion [3]. This section will mostly address primary adrenal malignancies.

### 9.1. Computed Tomography

**Figure 1:**
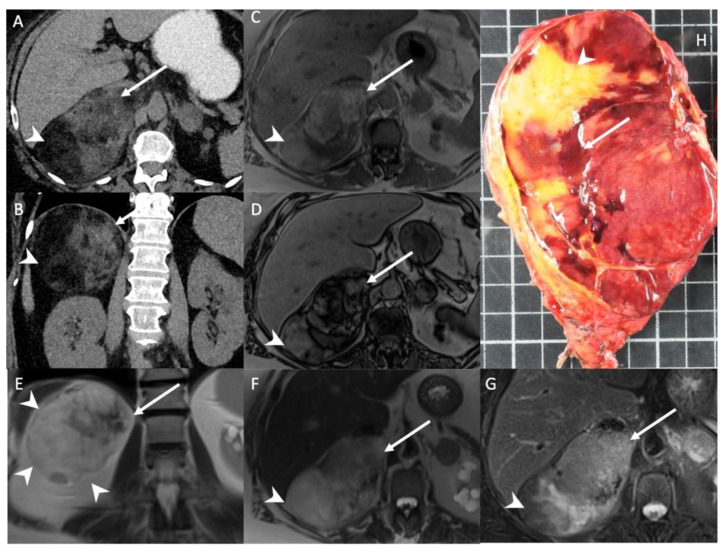
A 56-year-old woman with right adrenal incidentaloma. Unenhanced CT images in the axial (**A**) and coronal (**B**) planes show a 12-cm, well limited and encapsulated adrenal lesion, with low-attenuation (−70 HU) areas consistent with macroscopic fat content (arrowheads) and high-attenuation (30 HU) areas (arrows) consistent with hematopoietic tissue. T1-weighted in-phase (**C**) and out-of-phase (**D**) images in the axial plane show the area with heterogeneous signal drop (arrow) in the out-of-phase image. Of note, no signal drop is seen in the areas containing macroscopic fat (arrowhead). T2-weighted images in the coronal (**E**) and axial (**F**) planes show internal macroscopic fat (arrowheads) with a signal intensity similar to that of retroperitoneal fat, whereas the hematopoietic tissue (arrow) is hypointense. (**G**) The fat-suppressed T2-weighted image in the axial plane shows a drop in signal intensity in the macroscopic fat portion (arrowhead) by comparison with the signal intensity in (**F**), but not in the hematopoietic tissue (arrow). (**H**) Photograph showing gross specimen after right adrenalectomy. Pathologic analysis confirmed a well-encapsulated myelolipoma containing both red hematopoietic tissue (arrow) and yellow macroscopic fat (arrowhead). Each square in the background has a side of 1 cm.

**Figure 2:**
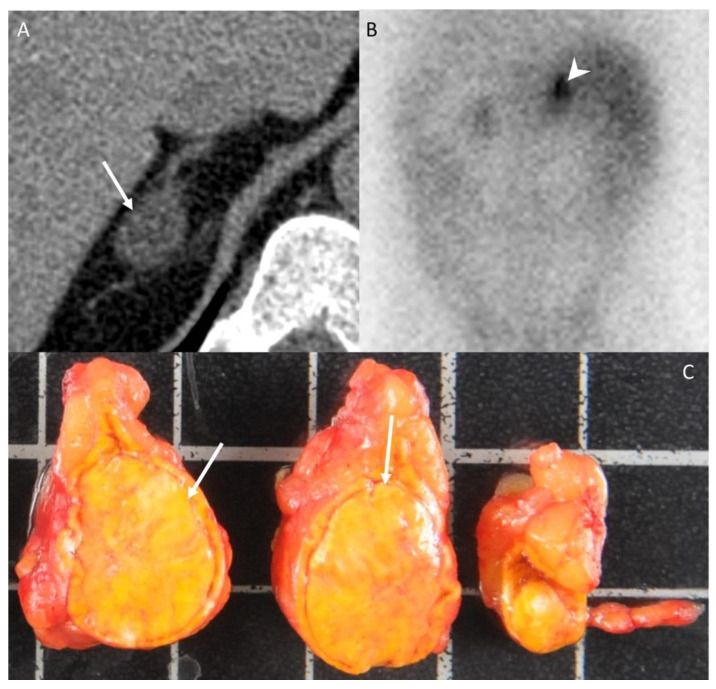
A 42-year-old woman without any medical history presenting with arterial hypertension. (**A**) Unenhanced CT image in the axial plane showing a 22-mm, homogeneous, right adrenal nodule (arrow) with an attenuation value of −6 HU, consistent with typical adrenal adenoma. (**B**) The 131I-iodo-cholesterol scintigraphy image in the posteroanterior projection shows a marked uptake of 131I-iodo-cholesterol by the right adrenal gland (arrowhead) consistent with a secreting adenoma. (**C**) Photograph showing the gross specimen after right adrenalectomy. Pathologic analysis confirmed a typical, homogeneous, well-encapsulated, benign adrenal adenoma (arrows) with a Weiss score of 0 and no other localization. The patients had no recurrence 10 years after the diagnosis. Each square in the background has a side of 1 cm.

**Figure 3:**
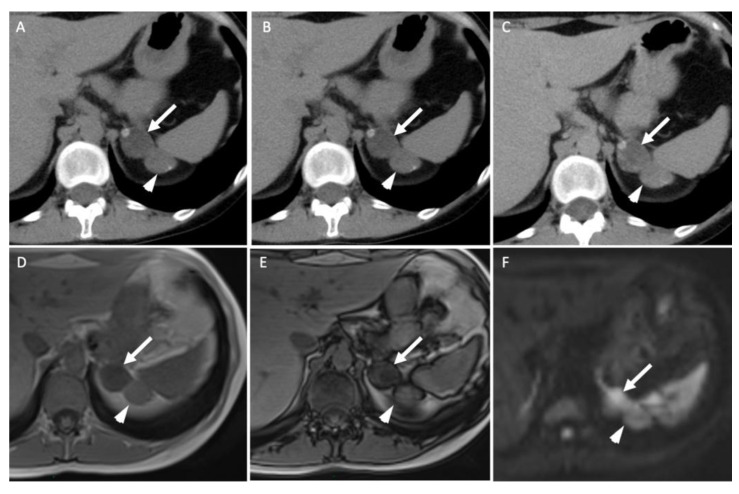
A 45-year-old woman with ACTH-independent Cushing syndrome. Unenhanced (**A**) and contrast-enhanced CT images obtained at 60 s (**B**) and 10 min (**C**) after intravenous administration of iodinated contrast material in the axial plane show two adrenal nodules in the left adrenal gland. The anterior nodule (arrows) has attenuation values of −5 HU in the unenhanced image, 50 HU at 60 s and 17 HU at 10 min; the absolute washout is 0.69 and the relative washout is 0.66. The posterior nodule (arrowheads) has attenuation values of 20 HU, 80 HU and 50 HU; the absolute washout is 0.50 and the relative washout is 0.38, which are not associated with a typical benign adrenal lesion. Unenhanced T1-weighted in-phase (**D**) and out-of-phase (**E**) MR images in the axial plane show a homogenous signal drop in the anterior nodule (arrow) in the out-of-phase image in comparison with the in-phase image and no signal drop in the posterior one (arrowhead). (**F**) The diffusion-weighted MR image (b = 1000 s/mm^2^) in the axial plane reveals two adrenal nodules (arrow/arrowhead) with a signal intensity similar to that of the spleen. Because of the secreting syndrome and atypical imaging features, the patient had an “en bloc” resection. Pathological analysis revealed two benign adrenal adenomas with Weiss histopathological scores of 1 for the anterior nodule and 2 for the posterior one.

CT is the most widely used imaging modality for the investigation of adrenal lesions [40]. Conventional CT examinations must include an unenhanced acquisition. Lipid-rich AA, spontaneous hematoma [41], myelolipoma and cysts can be diagnosed with CT, with almost 100% specificity (Figure 1, Figure 2 and Figure 3) [18,19]. Other situations require intravenous administration of an iodinated contrast material to calculate the APW and RPW [18]. 

**Figure 4:**
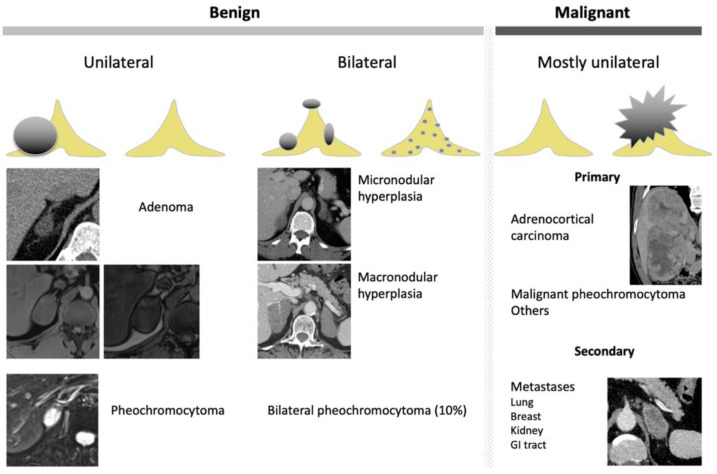
Graphical summary of benign and malignant adrenal incidentaloma on CT and MRI. GI, gastrointestinal.

Nevertheless, the precise characterization of other adrenal lesions remains difficult using CT alone, with a significant degree of overlap in CT features between the different non-AA lesions (Figure 4). ACC presents as a large adrenal lesion (97% of ACCs are >4 cm), with non-contrast attenuation values of >20 HU, possible necrosis, calcification and hemorrhagic patterns but without specific features [26,42,43]. A venous extension is a classical pattern that is rarely encountered [42,43]. Similarly, for right-sided ACCs, bulges and contour disruption on CT and MRI are associated with liver invasion and are consistent with malignancy [44]. Mesenchymal tumors (i.e., neurofibroma and schwannoma) have solid portions with a fibrotic pattern that may show progressive enhancement without washout [45]. Finally, adrenal metastasis presents as atypical adrenal lesion with increasing dimensions over time during the follow-up [3,46]. Adrenal metastases may present features similar to the primitive tumors, such as macroscopic fat for hepatocarcinoma or liposarcoma metastases [46]. 

**Figure 5:**
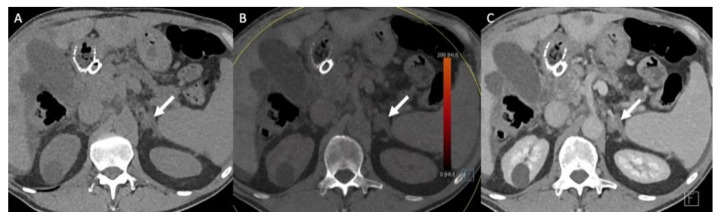
A 70-year-old man with a left adrenal lipid-poor adenoma known for 12 years without any changes in the imaging patterns (arrows). (**A**) The true unenhanced dual– CT image in the axial plane shows the left adrenal nodule (arrow) with a mean attenuation value of 13 HU. (**B**) In the virtual unenhanced (i.e., virtual non-contrast) dual-energy CT image in the axial plane, the adenoma (arrow) has a mean attenuation value of 14 HU, which is similar to the mean attenuation value obtained with the true unenhanced CT image. (**C**) In the venous phase CT image, the adrenal adenoma (arrow) has a mean attenuation value of 59 HU.

Dual-energy CT (either single or dual-source) allows the generation of virtual non-contrast (VNC) images, monochromatic images and material density images [47,48]. The attenuation values obtained with VNC images correlate with those obtained in true unenhanced images, but there is some degree of discordance (in the range of 5–10 HU), thus limiting the adoption of the usual ±10 HU threshold for characterization purposes (Figure 5) [49,50]. 

For the diagnosis of lipid-rich AA, VNC using the usual cut-off of 10 HU using an acquisition performed between 70 and 100 s after intravenous administration of iodinated material yields 71% sensitivity, similar to single-phase CT [51]. Conversely, an early acquisition (60 s) may result in an overestimation of the VNC attenuation values [51]. For differentiating between AA and adrenal metastases, the mean iodine density-to-VNC ratio is greater in AA than in metastases because of their maximal iodine uptake during the portal phase (14.5 vs. 4.6; *p* < 0.001) [49]. This ratio has better diagnosis performance than any other dual-CT features alone (area under the ROC curve (AUROC): 0.98 (95% CI: 0.95–1.00) vs. 0.91 (95% CI: 0.85–0.95) for VNC alone and 0.79 (95% CI: 0.72–0.85) for iodine density alone; *p* < 0.001) using a threshold of ≥6.7 [49]. 

Photon counting CT is a new technology that provides high-resolution images, a high contrast-to-noise ratio and reduced electronic noise [52,53]. In addition, it helps obtain VNC images from contrast-enhanced images, thus reducing radiation exposure [54]. Application to the abdomen is currently ongoing but no studies have reported results for adrenal gland characterization so far [55]. 

### 9.2. Magnetic Resonance Imaging

MRI is used as a second-line imaging modality for the characterization of adrenal lesions [40]. It may help diagnose adenomas with non-contrast attenuation values between 10 and 30 HU, when a calculation of the APW and/or RPW cannot be performed [56,57]. The standard MRI protocol includes unenhanced T1- and T2-weighted sequences and T1- or T2-weighted sequences with fat suppression to identify macroscopic fat content consistent with myelolipoma and internal liquid consistent with adrenal cysts [19]. Chemical shift sequences that combine in-phase (IP) and out-of-phase (OP) T1-weighted sequences (or DIXON sequences) help depict intracellular fat [58]. Intracellular lipids induce a drop in signal intensity in OP images in comparison with IP images. An intense and homogeneous signal drop in OP compared with IP is accurate for the diagnosis of AA [56,57]. Evaluation of the lipid content can be performed qualitatively or quantitatively. Different ratios have been described; however, a meta-analysis found no significant differences between visual evaluations of the chemical shift images and quantitative analyses [59]. Moreover, the addition of dynamic contrast-enhanced MRI sequences to chemical shift imaging improves the diagnostic capabilities of chemical shift imaging alone, with 94% sensitivity and 98% specificity versus 87% sensitivity and 95% specificity for standalone chemical shift imaging for the diagnosis of AA [59,60,61]. DWI has limited capacity for characterizing adrenal lesions and differentiating between benign and adrenal lesions [62]. Chemical shift imaging was found to be more accurate than DWI in a large prospective study for differentiating between lipid-poor AA and metastases in patients with known cancer [63].

Magnetic resonance spectroscopy (MRS) analyzes the resonance spectrum of protons that change according to the molecular environment. Adrenal MRS mostly consists of the analysis of 1-hydrogen. A high choline peak strongly suggests malignancy [61]. A metabolic choline/creatinine ratio of >1.2 has 100% sensitivity and 98.2% specificity for the diagnosis of benign (i.e., AA or pheochromocytoma) vs. malignant (i.e., ACC or adrenal metastasis) adrenal lesions [64]. Pheochromocytoma has a specific catecholamine signature spectrum [65]. Similarly, paragangliomas induced by mutations in the gene encoding mitochondrial succinate dehydrogenase (SDHx/SDHx) have a specific succinate spectrum signature, detected at 2.44 ppm on MRS in all biallelic mutated patients [66]. One limitation to the routine use of MRS is the duration of acquisition of the spectroscopy sequence. 

## 10. Nuclear Medicine and Radiotracers

### 10.1. ^18^F-FDG PET/CT 

**Figure 6:**
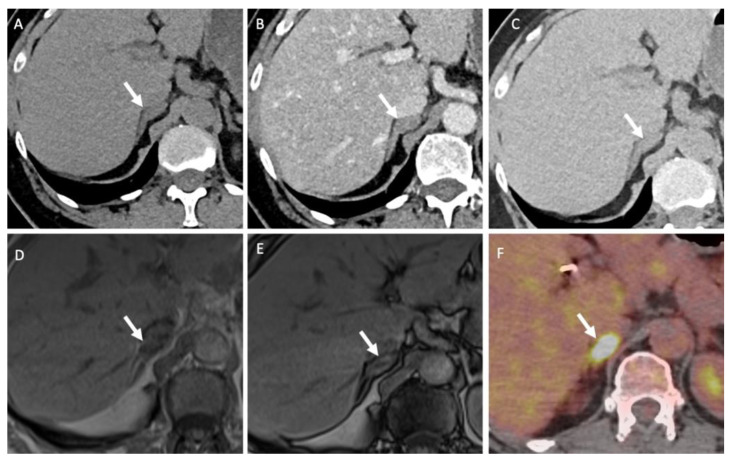
A 51-year-old man with a right adrenal nodule discovered during the initial staging of skin melanoma. Unenhanced (**A**) and contrast-enhanced CT images obtained at 60 s (**B**) and 10 min (**C**) after intravenous administration of iodinated contrast material in the axial plane show a nodule (arrows) in the right adrenal body. The nodule has attenuation values of 30 HU in the unenhanced image, 55 HU at 60 s and 40 HU at 10 min; the absolute washout is 0.60 and the relative washout is 0.27, which are not associated with a typical benign adrenal lesion. The unenhanced T1-weighted in phase (**D**) and out-of-phase (**E**) MR images in the axial plane show no signal drop of the nodule (arrows) in the out-of-phase image. (**F**) The ^18^F-FDG PET/CT image in the axial plane shows marked uptake of ^18^F-FDG by the right adrenal nodule. The nodule has a maximal standardized uptake value (SUVmax) of 8.4 and a liver-to-adrenal ratio of 3.8. Histopathologic analysis of a biopsy specimen obtained from percutaneous biopsy confirmed melanoma metastasis to the right adrenal gland.

**Figure 7:**
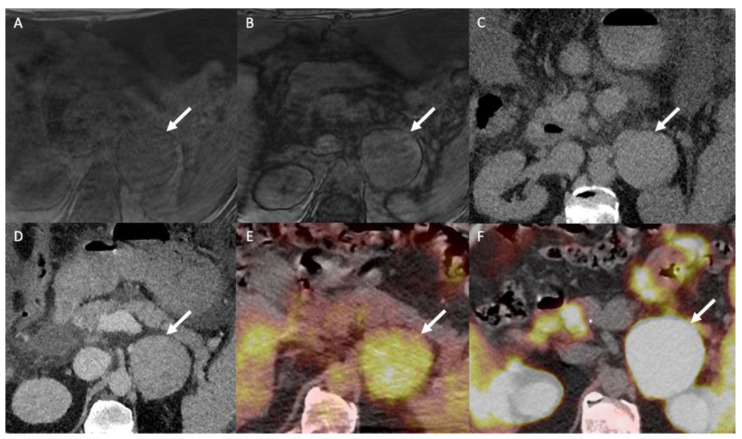
A 60-year-old man with multifocal hepatocellular carcinoma and a left adrenal mass. The T1-weighted in-phase (**A**) and out-of-phase (**B**) images in the axial plane show an enlarged left adrenal gland (arrow) without a signal drop between the in-phase (signal intensity = 243) and out-of-phase (signal intensity = 236) images, consistent with absent intracellular fat content. (**C**) The unenhanced CT image in the axial plane reveals a 66-mm, well limited and homogeneous left adrenal mass (arrow), with a mean attenuation value of 31 Hounsfield units (HU). (**D**) The contrast-enhanced CT images obtained at 60 s after intravenous administration of iodinated contrast material in the axial plane show a homogeneously enhanced adrenal mass with a mean attenuation value of 55 HU. The mean attenuation values were 48 HU at 60 s, 40 HU at 5 min and 38 HU at 10 min. The absolute washout percentage was 59% and the relative washout percentage was 21%. These findings are not typical of a benign adrenal mass and are consistent with a malignant adrenal lesion. (**E**) The ^18^F-FDG PET/CT image in the axial plane shows increased uptake of ^18^F-FDG by the left adrenal mass (arrow), with a SUVmax of 5.2. The SUVmax of hepatic parenchyma was 4.8, resulting in a liver to adrenal ratio of 1.1. (**F**) The ^18^F-choline PET/CT image in the axial plane shows an increased uptake of ^18^F-FDG by the left adrenal gland with a SUVmax of 14.8. ^18^F-choline is a radio tracer that shows high uptake by hepatocellular carcinoma. The discordance between the low ^18^F-FDG uptake and the marked ^18^F-choline uptake is common for poorly differentiated hepatocellular carcinoma metastases. Histopathological analysis of the tissue sample obtained by percutaneous biopsy confirmed adrenal metastasis from hepatocellular carcinoma.

^18^F-FDG PET/CT is a non-specific examination indicated for differentiating between benign and malignant atypical adrenal lesions. The adrenal-to-liver maximum SUV ratio, rather than the adrenal maximum SUV (SUV_max_) alone, helps in discriminating between benign and malignant adrenal lesions: a SUV_max_ cut-off of 3.4 has 100% sensitivity (95% CI: 85–100%) and 70% specificity (95% CI: 54–83%) for a diagnosis of malignant vs. benign adrenocortical lesions; a liver-to-adrenal SUV_max_ ratio cut-off of 1.45 increases the specificity to 88% (95% CI: 75–96%) while keeping a sensitivity of 100% (95% CI: 85–100%) for discriminating between ACC and AA [67]. ACC shows greater ^18^F-FDG uptake than AA, and ^18^F-FDG uptake correlates with the histopathological Weiss score and patient prognosis [67]. The ^18^F-FDG metabolic tumor volume is a prognostic factor that correlates with outcome in patients with ACC prior to any treatment or in patients with tumor recurrence [68]. In the specific context of patients with known cancer, ^18^F-FDG-PET helps differentiate between metastases and lipid-poor AA using a liver-to-adrenal SUV_max_ ratio cut-off of 2.5 (Figure 6 and Figure 7) [69]. However, the main limitation of ^18^F-FDG PET/CT is the poor specificity when the pre-test probability is low. This is especially true for myelolipoma, which may display marked but non-specific ^18^F-FDG uptake [70].

### 10.2. 131I-6β-Iodomethyl-19-norcholesterol (Noriodocholesterol)

131I-6β-iodomethyl-19-norcholesterol (noriodocholesterol) is a cholesterol analog-labeled compound that accumulates in the adrenocortical gland under the influence of ACTH (Figure 4) [71]. Adrenocortical scintigraphy with noriodocholesterol has 100% accuracy for the diagnosis of large AA (>15 mm) and can depict preclinical functioning AA. Unfortunately, this examination requires complex logistics to obtain the very short half-life radiotracer and is difficult to implement in daily practice [71].

### 10.3. Metonidate 

Metonidate (MTO) is an inhibitor of aldosterone reaction chain synthesis. It can be labeled with ^123-^iodine for single photon emission computed tomography (SPECT) or with ^11^C for PET imaging. ^11^C-metomidate PET/CT has 86% sensitivity and 96% specificity for the diagnosis of adrenocortical vs. non-adrenocortical lesions [72]. One limitation is its 20-min half-life, which limits further clinical development. MTO-labeled compounds can be used for targeted radionuclide therapy in patients with advanced ACCs for whom no other options are available [73]. ^11^C-MTO may also be used as a non-invasive alternative to selective adrenal vein sampling to lateralize lesions in patients with primary aldosteronism due to AAs of <1 cm [74]. 

### 10.4. ^123-^Iodine Labeled Meta-Iodobenzylguanidine (123I-MIBG) 

^123-^Iodine labeled meta-iodobenzylguanidine (123I-MIBG) has a structural analogous to norepinephrine, and has high sensitivity (83–100%) and specificity (95–100%) for the diagnosis of pheochromocytoma [75]. However, despite the high specificity, the sensitivity is only 52–75% for the detection of extra-adrenal, multiple, recurrent and hereditary paraganglioma. There are possible false positive findings due to physiological visualization of the adrenal medulla [76]. Even in patients at risk for metastatic disease, ^123^I-MIBG has no benefit in comparison with the combination of CT/MRI in the preoperative workup of patients with pheochromocytoma. Hence, the role of ^123^I-MIBG for clinical decision-making remains limited [76,77].

### 10.5. ^18^F-FDOPA and 68Ga-DOTA-Somatostin Analogs (SSA)

**Figure 8:**
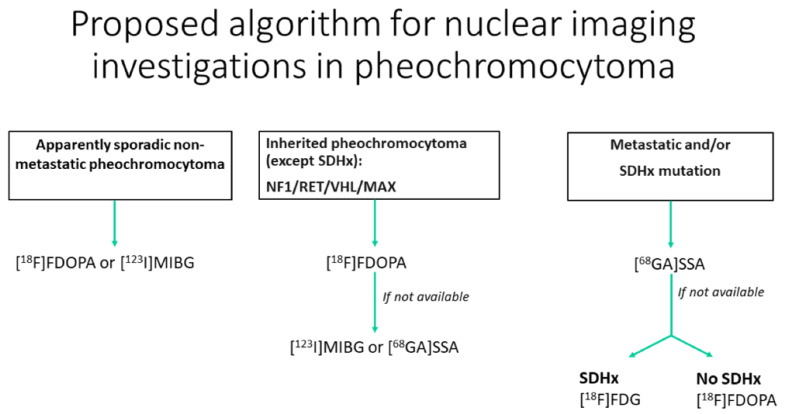
Diagram of the algorithmic approach for the appropriate use of nuclear medicine when adrenal pheochromocytoma is suspected (adapted from Taieb et al. [79]).

^18^F-FDOPA and 68Ga-DOTA-somatostin analogs (SSA) are, respectively, a tracer of the catecholamine metabolic pathway and an analog of the somatostatin available for the characterization of pheochromocytomas. ^18^F-FDOPA has ≥90% sensitivity and 95–100% specificity for the diagnosis of pheochromocytoma, with fewer practical constraints and no drug interactions, unlike ^123^I-MIBG [78]]. In succinate dehydrogenase-B (SDHB)-negative patients, ^18^F-FDOPA has 93% sensitivity compared with 20% in SDHB-positive patients [79]. ^68^Ga-DOTA-SSA is more sensitive in patients with SDH-B mutations than in those without mutations, with a 98.6% overall detection rate [80]. Recent guidelines advocate the use of ^18^F-DOPA PET for inherited pheochromocytoma (NF1/RET/VHL/MAX) except for SDH-mutated (SDHx) patients. However, the use of ^18^F-FDOPA is not approved in the USA. For metastatic paraganglioma, ^68^Ga-DOTA-SSA should be preferred, regardless of the genetic background (Figure 8) [75]. ^68^Ga-SST targeting somatostatin receptor 2 (SSTR2) shows promising results for the detection of pheochromocytoma and paraganglioma [81]. It can be considered as a theranostic approach, integrating imaging and therapy with ^177^lutetium-DOTATATE as the future of the therapeutic arsenal for metastatic pheochromocytoma/paraganglioma [81]. 

^18^F-DOPA, ^18^F-fluoro-labeled fluorodopamine; ^123^I-MIBG, ^123-^iodine-labeled meta-iodobenzylguanidine; ^68^GA-DOTA-SSA, 68-gallium labeled DOTA somatostatin analogs; SDHx, succinate dehydrogenase Type x; NF1, neurofibromatosis type 1; RET, RET proto-oncogene; VHL, von Hippel–Lindau; MAX, Myc-associated factor X.

### 10.6. Other Radiotracers That May Be Useful for Adrenal Functional Imaging 

^68^Gallium prostate membrane-specific antigen (^68^GaPMSA) uptake by the solid portion of adrenal schwannoma may be important but remains poorly studied [82]. However, because of the rarity of these adrenal lesions, its role may be limited to the fortuitous detection of schwannomas when ^68^GaPMSA is used for another indication, such as prost ate cancer workup. Similarly, for the characterization of atypical myelolipoma, ^99m^Tc-sulfur-colloid scintigraphy is a specific radiotracer of bone marrow but is seldom used in daily practice [83].

## 11. Adrenal Collision Tumors

Collision tumor consist of an association of two tumor types in two adjacent adrenal lesions. Two benign, one benign and one malignant, or two malignant histological subtypes may be associated [84]. In adrenal glands, many occurrences of the association of AA with pheochromocytoma, myelolipoma, metastases or ACC have been reported. Adrenal collision tumors can be undetected if all components of the entire adrenal lesion are not carefully analyzed. However, the performance of non-invasive imaging in the identification of adrenal collision tumors has not been formally evaluated (Figure 3) [84]. 

## 12. Minimally Invasive Radiological Methods for the Characterization of Adrenal Lesions

### 12.1. Adrenal Vein Sampling

**Figure 9:**
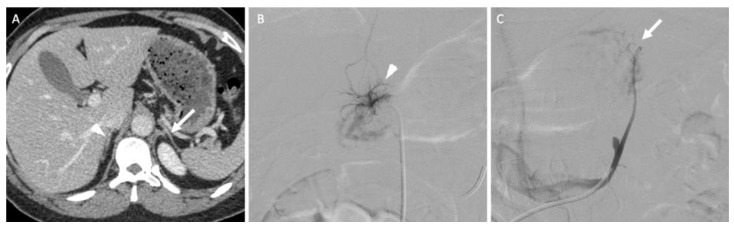
A 45-year-old woman with primary hyperaldosteronism with normal adrenal glands on computed tomography (CT) and MRI who underwent bilateral adrenal vein sampling. (**A**) The CT image in the axial plane obtained 70 s after intravenous administration of iodinated contrast material shows two thin adrenal glands (the arrow indicates the left adrenal gland; the arrowhead indicates the right adrenal gland) without a nodular portion. (**B**) The digital subtracted angiographic image shows selective catheterization of the right adrenal vein (arrowhead) using a 5-F Shepherd hook catheter. (**C**) The digital subtracted angiographic image shows selective catheterization of the left adrenal vein (arrow) using a 5-F left renal catheter. Blood sample analysis confirmed elevated aldosterone secretion from the right adrenal gland. The patient underwent right adrenalectomy, and her aldosterone serum level returned to normal values.

In the specific situation of primary hyperaldosteronism, when no functional or morphological examinations can identify the responsible adrenal gland, bilateral adrenal vein sampling (AVS) is an effective option that conveys high diagnostic accuracy and a low complication rate for trained interventional radiologists (Figure 9) [38]. Some authors recommend the systematic use of AVS except for patients under the age of 35 years with a florid primary hyperaldosteronism when a surgical option is considered because non-functioning adrenal lesions are frequent, but this remains debated and associated with a poor level of evidence [38]. 

AVS consists of femoral venous access to the adrenal veins to sample blood as close as possible from its point of secretion. The ratio of hormones between both glands allows the lateralization of the secretion at a level of >4 for aldosterone [38]. Pharmacological stimulation with a continuous cosyntropin infusion may be used, although no evidence suggests a better outcome compared with unstimulated adrenal vein sampling [38]. 

### 12.2. Percutaneous Biopsy

Histopathological confirmation using percutaneous biopsy may be required to avoid surgery only in specific situations, such as undetermined, non-secreting adrenal lesions confirmed using non-invasive tools, suspected metastasis in a patient with a cancer for which the presence of an adrenal metastasis would alter management, searching for specific mutations for further targeted therapies and a suspicion of rare tumors such as adrenal sarcoma, primary lymphoma or granulomatosis [1,85]. Undetermined adrenal lesions or suspected ACC are contraindications for adrenal biopsy [1,85]. Similarly, in all situations, functional pheochromocytoma must be ruled out and, if suspected, specific intensive care management must be performed. When performed, percutaneous biopsy is safe, with a pooled overall complication rate of 2.5% (including pneumothorax, hypertensive episodes or hematoma) and effective, with a pooled non-diagnostic rate of 8.7% [86]. 

## 13. New Technologies for Adrenal Lesion Characterization

### 13.1. Principles and Definitions

Artificial intelligence (AI) may provide added value to computed image analysis. It requires different steps from image acquisition for the analysis. The first step mostly consists of lesion segmentation. Segmentation aims to extract the volume of interest from the entire data. The segmentation may be manual or automated using different algorithms such as multi-organ atlas-based, landmark-based, shape model-based and neural network-based algorithms [87]. Despite extensive literature on automatic segmentation in the retroperitoneum such as pancreatic lesions [86], there is no specific literature about adrenal gland and adrenal lesion segmentation, and no automatic algorithm exists yet [87]. 

After segmentation, the analysis may be performed using several algorithms and strategies, including machine learning (ML) and deep learning (DL). ML refers to providing more data to continuously update the computer/software performance for a given task. This implies that the computer/software learns from the data and improves its performance through experience [88]. DL refers to the use of a layered structure of algorithms (i.e., the deep neural network), which is supposed to imitate human perception in recognizing features from the input data [88]. DL can be considered as the most advanced subcategory of ML. 

Midway between AI and morphological imaging, texture and radiomics analyses are quantitative methods that provide measurements of heterogeneity using parameters based on local variations in image voxel brightness that are often beyond the reach of the human eye [89]. Extracted features consist of first-order features (e.g., histograms and shape) that analyze the voxel values, whatever their location, and second-order features that take the spatial location and interrelationships between voxels into account by using matrixial transformation of the voxels [90]. The most frequently used matrix is the grey level co-occurrence matrix [90]. For each matrix, statistical features of heterogeneity may be extracted and, ultimately, hundreds of parameters can be extracted [90]. They allow the development of quantitative scores or AI algorithms for differentiating benign from malignant adrenal lesions, detecting some histological subtypes or developing some prognostic biomarkers when validated [91,92,93,94]. The goal of radiomics is to extract biomarkers predicting survival or response to treatments from pre-operative non-invasive imaging similar to those that can been obtained invasively with transcriptomics or genomics after surgery [95]. 

### 13.2. Current Results and Future Applications

To date, clinical applications of AI remain limited in daily practice and most studies have a low level of evidence. However, radiomic features may provide new tools for the diagnosis of atypical adrenal lesions and research applications. To date, AI tools mostly aim to differentiate benign from malignant adrenal lesions. Specific characterization remains in the field of research.

### 13.3. AI for the Diagnosis of Benign Adrenal Lesions

With first-order features, a >10% cut-off of negative voxels in a histogram derived from unenhanced CT data had 92.3% sensitivity and 100% specificity for the diagnosis of lipid-poor AA [96]. The histogram entropy yielded an AUC of 0.65 for differentiating benign from malignant adrenal lesions [97]. Second-order features may also help. Using more complex models, Ho et al. found 21 second-order features extracted from contrast-enhanced CT data that allowed better differentiation of lipid-poor AA from malignant adrenal lesions than the standard morphological features (AUCs of 0.8 vs. 0.6, respectively) [93]. Similarly, Stanzione et al. proposed a ML algorithm for differentiating between benign and malignant adrenal lesions with 91% accuracy [98]. 

For differentiating between lipid-poor AA and pheochromocytoma, four texture features extracted from unenhanced CT data had 96.6% sensitivity, 81% specificity and 85.2% accuracy for the diagnosis of pheochromocytoma versus lipid-poor AA [88]. ML algorithms including texture parameters had an AUC > 0.95 in a validation cohort and had performances similar to those of an experienced radiologist for correct classification of AA, lipid-poor AA and non-adenoma lesions (73% vs. 80%, respectively; *p* = 0.39) [91,92]. In the same study, a radiological score created from texture parameters yielded 86.2% sensitivity, 97.5% specificity, 94.4% accuracy and an AUC of 0.952 for the diagnosis of pheochromocytoma vs. lipid-poor AA [91]. 

In histogram analyses of an MRI ADC map using two diffusion coefficients (*b* = 0 and 800 mm^2^/s), pheochromocytomas have greater variance and entropy (0.13 ± 0.10 and 2.81 ± 0.22, respectively) than AAs (0.03 ± 0.02 vs. 2.37 ± 0.26, respectively) (*p* < 0.001 for both) [99]. A cut-off of 2.67 for entropy had 95% sensitivity and 92% specificity for the diagnosis of pheochromocytoma vs. AA (*p* < 0.001) [99]. 

Nakajo et al. found that homogeneity and entropy in ^18^F-FDG PET uptake are significantly different between lipid-poor AA (0.17 ± 0.05 vs. 6.52 ± 0.98, respectively) and pheochromocytoma (0.23 ± 0.07 vs. 6.47 ± 0.71, respectively) (*p* = 0.038 and 0.016, respectively) [100]. 

### 13.4. AI for the Diagnosis of Adrenal Malignancy

Regarding ACC, texture analysis has been poorly studied, with conflicting results. In one study, shape elongation and flatness, which are two first-order texture parameters, helped differentiate between ACCs with Ki67 ≤ 10 and those with Ki67 > 10 (AUCs of 0.70 and 0.78, respectively) [94]]. One major limitation to extensively studying ACC is the rarity of this tumor. 

In functional imaging, texture analysis extracted from ^18^F-FDG-PET/CT data did not prove superior to SUV_max_ alone for discriminating between AA and adrenal metastasis, as SUV_max_ alone has 86% sensitivity and 80% specificity in that task [100].

Some texture parameters derived from ^18^F-FDG PET/CT data, such as the non-uniformity calculated on the grey- level run length matrix, can be used as a predictor of overall survival in patients with primary adrenal lymphoma [101].

### 13.5. Future Applications

As previously described, most of the existing AI tools suffer from a lack of level of evidence and applicability for use in daily practice. The next steps consist of selection of the tools and validation in large prospective cohorts. Researchers may also consider using AI-extracted imaging features in association with clinico-biological features as previously performed with conventional imaging to improve the capacity for adrenal lesion characterization [26]. 

## 14. Conclusions

In conclusion, adrenal lesions have currently a prevalence of 4–6%, but it can be assumed that these figures may increase with the rise in the use of abdominal imaging examinations, along with improvements in imaging technology. Although AA remains the most frequent adrenal lesion, many histological subtypes exist, with possible heterogeneous presentations within conventional imaging, resulting in limited specificity and accuracy. Radiologists must be aware of typical features to avoid overdiagnosis of benign or indolent lesions. Atypical features require a multidisciplinary approach with a comprehensive evaluation. Recent studies evaluating the capabilities of radiomics and artificial intelligence have shown encouraging results. but further research remains to be carried out to proceed further in the non-invasive characterization of adrenal gland lesions.

## Figures and Tables

**Table 1:** Simplified classification of frequent adrenal hyperplasia etiologies.

Non-Secreting Disease (60%)		Secreting Syndrome (40%)	
Adrenal disease	Systemic disease	ACTH-dependent (85%)	ACTH-independent (15%)
Hematoma	Metastasis	Pituitary adenoma	Macronodular hyperplasia
Infection	Infiltrative disease	Ectopic secretion	Primary pigmented nodular adrenocortical disease

Note: ACTH, adrenocorticotropic hormone.

## Abbreviations

3D	Three-dimensional
AA	Adrenal adenoma
ACC	Adrenocortical carcinoma
ACTH	Adrenocorticotrophic hormone
ADC	Apparent diffusion coefficient
AH	Adrenal hyperplasia
AI	Artificial intelligence
APW	Absolute percentage of washout
AUC	Area under the curve
AUROC	Area under the receiving operator characteristic curve
CT	Computed tomography
DL	Deep learning
DWI	Diffusion-weighted imaging
FDG	Fluorodeoxyglucose
HU	Hounsfield unit
IP	In-phase
MIBG	Metaiodobenzylguanidine
ML	Machine learning
MRI	Magnetic resonance imaging
MRS	Magnetic resonance spectroscopy
NPV	Negative predictive value
OP	Out-of-phase
PET	Positron emission tomography
PPV	Positive predictive value
ROC	Receiving operator characteristics
RPW	Relative percentage of washout
SDH	Succinate dehydrogenase
SPECT	Single-photon emission computed tomography
SSA	Somatostatin analog
SUV	Standardized uptake value
